# The importance of intervertebral disc material model on the prediction of mechanical function of the cervical spine

**DOI:** 10.1186/s12891-021-04172-1

**Published:** 2021-04-02

**Authors:** Amin Komeili, Akbar Rasoulian, Fatemeh Moghaddam, Marwan El-Rich, Le Ping Li

**Affiliations:** 1 School of Engineering, University of Guelph, Guelph, Canada; 2 Healthcare Engineering Innovation Center, Department of Mechanical Engineering, Khalifa University, Abu Dhabi, United Arab Emirates; 3 Department of Mechanical and Manufacturing Engineering, University of Calgary, Calgary, Canada

## Abstract

**Background:**

Linear elastic, hyperelastic, and multiphasic material constitutive models are frequently used for spinal intervertebral disc simulations. While the characteristics of each model are known, their effect on spine mechanical response requires a careful investigation. The use of advanced material models may not be applicable when material constants are not available, model convergence is unlikely, and computational time is a concern. On the other hand, poor estimations of tissue’s mechanical response are likely if the spine model is oversimplified. In this study, discrepancies in load response introduced by material models will be investigated.

**Methods:**

Three fiber-reinforced C2-C3 disc models were developed with linear elastic, hyperelastic, and biphasic behaviors. Three different loading modes were investigated: compression, flexion and extension in quasi-static and dynamic conditions. The deformed disc height, disc fluid pressure, range of motion, and stresses were compared.

**Results:**

Results indicated that the intervertebral disc material model has a strong effect on load-sharing and disc height change when compression and flexion were applied. The predicted mechanical response of three models under extension had less discrepancy than its counterparts under flexion and compression. The fluid-solid interaction showed more relevance in dynamic than quasi-static loading conditions. The fiber-reinforced linear elastic and hyperelastic material models underestimated the load-sharing of the intervertebral disc annular collagen fibers.

**Conclusion:**

This study confirmed the central role of the disc fluid pressure in spinal load-sharing and highlighted loading conditions where linear elastic and hyperelastic models predicted energy distribution different than that of the biphasic model.

## Introduction

Among major spine injuries, cervical injuries could be life-altering as they might cause permanent loss of neural function. Cervical fractures are common in intensive recreational activities, such as American football and rugby [1, 2]. Experimental measurement provided invaluable insight into spine function to some extent, yet fails to quantify precisely other important parameters, such as strain/stress distribution under impact loads. Moreover, empirical investigations of some *what-if* questions would be time- and cost-expensive. Simulation modelling proved to be a viable means in biomechanical studies, in vivo or otherwise.

The spine simulation models have been improved extensively using calibrated constitutive models [3–7], detailed geometry reconstruction [8–11], and realistic physiological loading measurements [12–14]. While there is a general agreement on modelling the bony parts, various material models for the intervertebral disc (IVD) and ligaments were shown to be valid under certain loading conditions. Effects of ligament material modelling on spine mechanical behavior have been investigated extensively [8, 15–18]. However, studies on spine biomechanical response obtained from different IVD material models are sparse [19]. The linear elastic (LE), hyperelastic (HE), nucleus cavity and multiphasic theories are the most commonly used constitutive theories for simulating nucleus pulposus (NP) and annulus fibrosus (AF). The annular collagen fibers were simulated using spring elements with an organized spatial distribution in concentric lamellae [20]. The LE theory is the simplest material model to apply but is incapable of describing the nonlinear mechanical behaviour of IVD [3, 21]. The HE constitutive models addressed nonlinear stress-strain behaviour [22, 23], but the energy conservation nature of the LE and HE models failed to simulate the IVD creep/relaxation behaviour caused by fluid-solid interaction [24]. Studies that used LE and HE models estimated the intradiscal pressure (IDP) with the disc hydrostatic stress [25, 26]. The cavity model assumed the NP as a fluid volume enclosed within a membrane [27–29]. Although this assumption represented the load-bearing characteristic of the IDP under high loading rates, it ignored the shear stresses and failed to model progressive disc deformations under cyclic loads. The multiphasic theory [30, 31] was the most recent material model that showed the evident link between the fluid and solid interactions [19, 32, 33]. The viscoelastic model would also simulate a rate-dependent mechanical response [34], yet it lacks an accurate prediction of some tissue biomechanical phenomena, such as tissue swelling and degeneration.

The effects of LE, HE, and multiphasic IVD material models on mechanical response were studied using simple axisymmetric models [35], however, it was shown that spine components, i.e. ligaments, endplates, and facets, have a considerable contribution to the mechanical response of the functional spinal unit (FSU) [8, 32, 35, 36]. Nowadays, the spine components are reconstructed from computer tomography scans or magnetic resonance images with a high spatial resolution to predict more precise load-sharing of the FSU and simulate in-vivo loading conditions. Flexion, extension, lateral bending and axial compression are typical loading conditions supported by the cervical spine during daily activities [37–41]. Range of motion (RoM), facets contact pressure, disc height change, IDP, and creep/relaxation were the most important mechanical parameters of the FSU studied in the literature. Therefore, in the present study, a detailed nonlinear 3D finite element (FE) model of C2-C3 FSU was presented with three different fiber-reinforced IVD material models, including LE, HE, and Biphasic theories. The objective of the present study was to compare the kinetics and kinematics predicted by these models under flexion/extension moments and cyclic compression load.

## Methodology

### FE meshes and material models

**Fig. 1:**
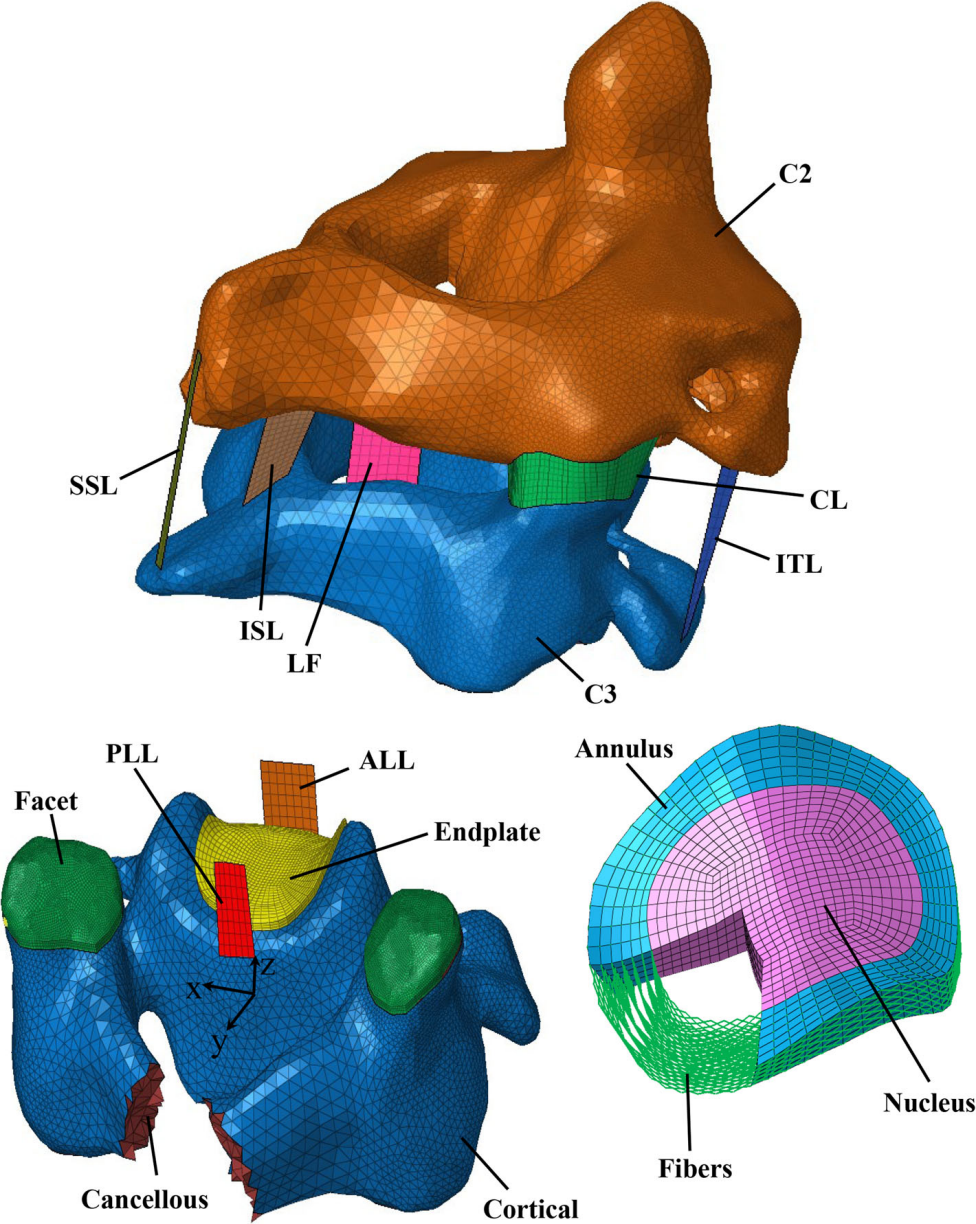
The simulated C2-C3 FSU components in the present study

A 3D FE model of the C2-C3 FSU was created (Fig. 1) and analyzed in Abaqus implicit software package (Dassault Systemes, 2017). Bony components geometry was reconstructed from 1 mm interval CT-Scans of a 39-year-old male [42]. These images were provided by the National Library of Medicine’s Visible Human Project (http://www.nlm.nih.gov/research/visible/visible_human.html). Each vertebra comprises cancellous and cortical bones, endplates, and articular facets (Fig. 1). The complex geometry of cortical bone was meshed using 3-node triangular shell elements of 1 mm thickness. Four-node tetrahedral solid elements were used to mesh the cancellous bone. Facet cartilages and endplates of 0.7 mm thickness were meshed with 8-node brick elements. Two capsules were created to connect the adjacent articular facets on both sides (Fig. 1) [22]. The IVD volume was divided into NP and AF ground substance with a proportion of 56 and 44%, respectively, according to the histological findings [43]. The AF mesh was generated by extruding seven layers of 8-node solid elements between two adjacent endplates, and reinforced in the circumferential direction by seven layers of collagen fibers (Fig. 1). Unidirectional nonlinear springs resisting tensile load only and organized in concentric lamellae with crosswise patterns close to ±35° were used to model the annular fibers [20]. The Anterior Longitudinal (ALL), Capsular (CL), Interspinous (ISL), Intertransverse (ITL), Ligamentum Flavum (LF), Posterior Longitudinal (PLL), and Supraspinous (SSL) ligaments were constructed using 4-node shell elements of 1 mm thickness by connecting their attachment points obtained from anatomical and histological studies to surrounding vertebrae [44–46].

Strain rate-dependent elasto-plastic Johnson-Cook material model was assigned to bony components [47, 48] (Table 1). The ligaments were defined by the Prony series viscoelastic model [47, 52, 53]. The LE material properties were assigned to the facet cartilages, and a frictionless surface-to-surface hard contact was defined between the two adjacent facets [9, 32] (Table 2). The effect of IVD material properties on the overall behaviour of FSU as well as the local spinal tissues was investigated by assigning LE, HE, and Biphasic material properties to the AF, NP and endplates (Table 3). All loads were applied to the centroid of C2, while the C3 bottom was completely fixed.

**Table 1:** Material properties of bony components for C2-C3 FSU model

Bony Components (Elasto-Plastic Model)			
**Material Properties**	**Cortical**	**Cancellous**	**Reference**
**Young Modulus,** ***E*** **(MPa)**	16,800	100	[49]
**Poisson Ratio,** ***ν***	0.3	0.29	
**Yield Stress,** ***a*** **(MPa)**	110	1.92	
**Hardening Modulus,** ***b*** **(MPa)**	100	20	[48]
**Hardening Exponent,** ***n***	0.1	1	
**Failure Plastic Strain,** ***ε***_***p***_ (**10**^***−*3**^**)**	9.86	14.5	[50]
**Maximum Stress (MPa)**	155	2.23	[51]
**Strain Rate Coefficient,** ***c***	1	1

**Table 2:** Material properties of ligaments, IVD collagen fibers, and facet cartilages for C2-C3 FSU model. *E* = Young Modulus, *ν* = Poisson Ratio

Ligaments (Prony Series Viscoelastic Model), ${\boldsymbol{g}}_{\boldsymbol{R}}=\mathbf{1}-{\sum}_{\boldsymbol{i}=\mathbf{1}}^{\mathbf{3}}{\boldsymbol{g}}_{\boldsymbol{i}}\left(\mathbf{1}-{\boldsymbol{e}}^{-\frac{\boldsymbol{t}}{{\boldsymbol{\tau}}_{\boldsymbol{i}}}}\right)$				
Prony Series Variables	*i* = 1	*i* = 2	*i* = 3	Reference
*τ*_*i*_	0.3991	0.3605	0.1082	[54]
*G*_*i*_	0.7	0.149	0.15	
*t*_*i*_	3.4519	2000	7000	
	**Material Properties**			
**Ligaments**	**ALL**	***E*** **(MPa)**	11.4	[55]
		***ν***	0.4	
	**CL**	***E*** **(MPa)**	7.7	[56]
		***ν***	0.4	
	**ISL**	***E*** **(MPa)**	4.7	[52, 57]
		***ν***	0.4	
	**ITL**	***E*** **(MPa)**	4.7	[52, 57]
		***ν***	0.4	
	**LF**	***E*** **(MPa)**	4.2	[58]
		***ν***	0.4	
	**PLL**	***E*** **(MPa)**	9.12	[58]
		***ν***	0.4	
**Collagen Fibers**	***E*** **(MPa)**		100	[20]
	***ν***		0.3	
**Facet Cartilage**	***E*** **(MPa)**		11	[32]
	***ν***		0.4

**Table 3:** Material properties of IVD components in the LE, HE, and Biphasic models of the C2-C3 FSU [35]

IVD (Biphasic Model)	NP			AF			Endplate		
Material Properties	LE	HE	Biphasic	LE	HE	Biphasic	LE	HE	Biphasic
**Young Modulus,** ***E*** **(MPa)**	1	–	1	2.5	–	2.5	20	–	20
**C10**		0.12			0.56				
**C01**	–	0.09	–	–	0.14	–	–	–	–
**D1**		1			1				
**Poisson Ratio,** ***ν***	0.1	–	0.1	0.1	–	0.1	0.1	–	0.1
**Saturated Permeability,** $\underset{\_}{\boldsymbol{k}}$ **(mm/s)**	–	–	8.4e-9	–	–	2.85e-9	–	–	6e-8
**Void Ratio**	–	–	4	–	–	2.33	–	–	4
**Specific Weight of Liquid,** ***γ*** **(N/m**^**3**^**)**	–	–	9810	–	–	9810	–	–	9810

### Loading conditions

#### Cyclic compression

A 200 N compression ramp in 20 s was applied to the centroid of C2 following the Skrzypiec et al. [59] loading condition, and the C3 vertebral body was fully fixed. The IVD stress predicted by the developed FE models was compared with experimental measurements [59]. Furthermore, the performances of LE, HE, and Biphasic models were compared under a periodical compressive load with an amplitude of 100 N and frequency of 0.5 Hz in the form of *F* = 50 + 50 cos(*π*(*t* − 1)). This loading range was shown to cover the approximate 46 ± 7 N weight of human head [60, 61]. The cyclic compression was applied for 11,000 cycles, which is in line with the estimated number of steps a soldier takes during a 19 km walk to gain the Expert Infantryman Badge [62].

#### Sagittal bending

Quasi-static bending moments with magnitudes of 1 and 1.5 Nm were applied to the centroid of C2 in the anterior (+ve x-axis) and posterior (−ve x-axis), representing flexion and extension in the sagittal plane, respectively. The 1–1.5 Nm flexion/extension moments were judged to be sufficient to produce physiologic motions in the actual intact conditions [38, 63]. This loading condition was applied to validate the model with experimental results [38, 63]. The effect of the IVD material model on the FSU load-sharing was studied under a flexion/extension moment of 1.5 Nm in 30 s. Strain Energy stored in the FSU components was used to investigate their load-sharing.

## Results

### Cyclic compression

**Fig. 2:**
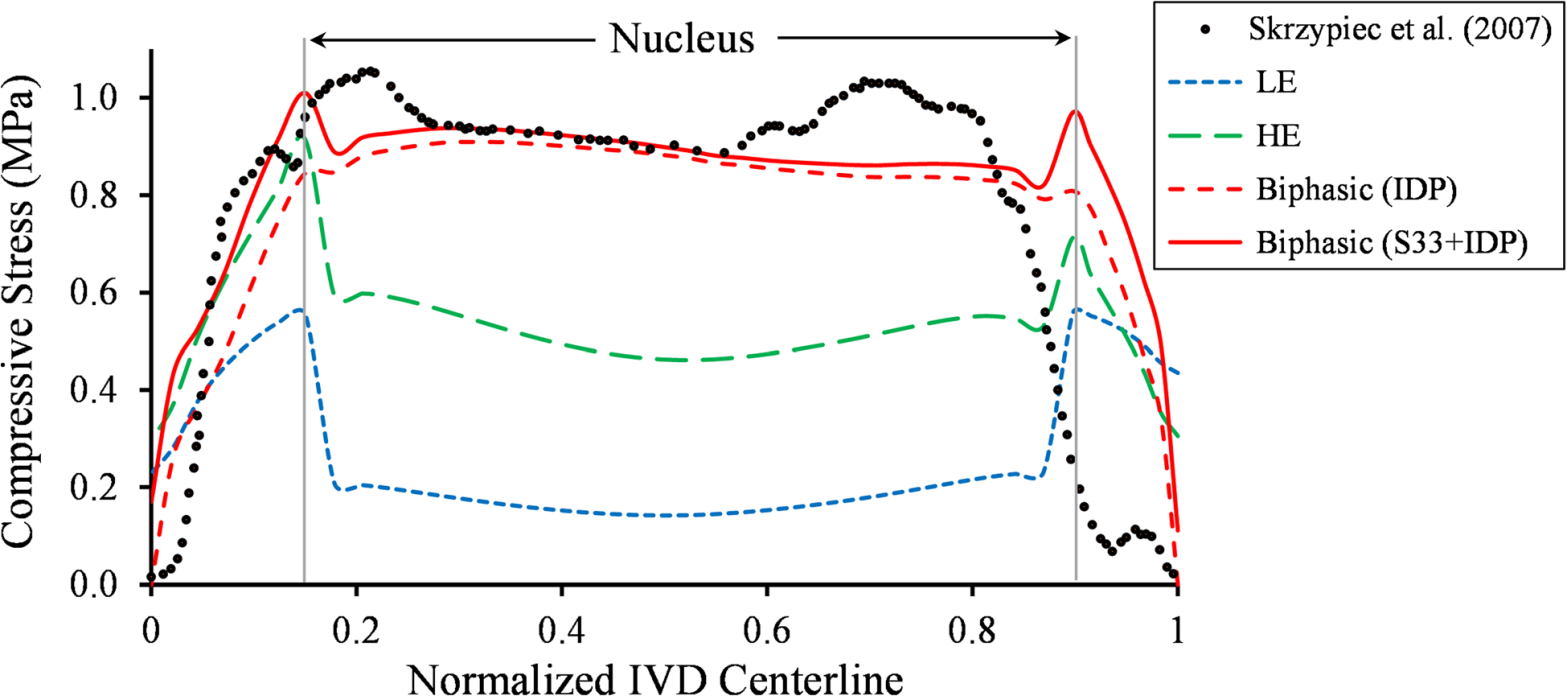
The IVD stress profile along the sagittal midline diameter of the disc. The results were compared with the digitized curve from Skrzypiec et al. study [59]. The x-axis is the normalized length of the centerline, where x = 0 and 1 representing the posterior and anterior AF, respectively. In LE and HE models, the hydrostatic stress represented the IDP stress. S33: the compressive stress in the direction of the applied load

**Fig. 3:**
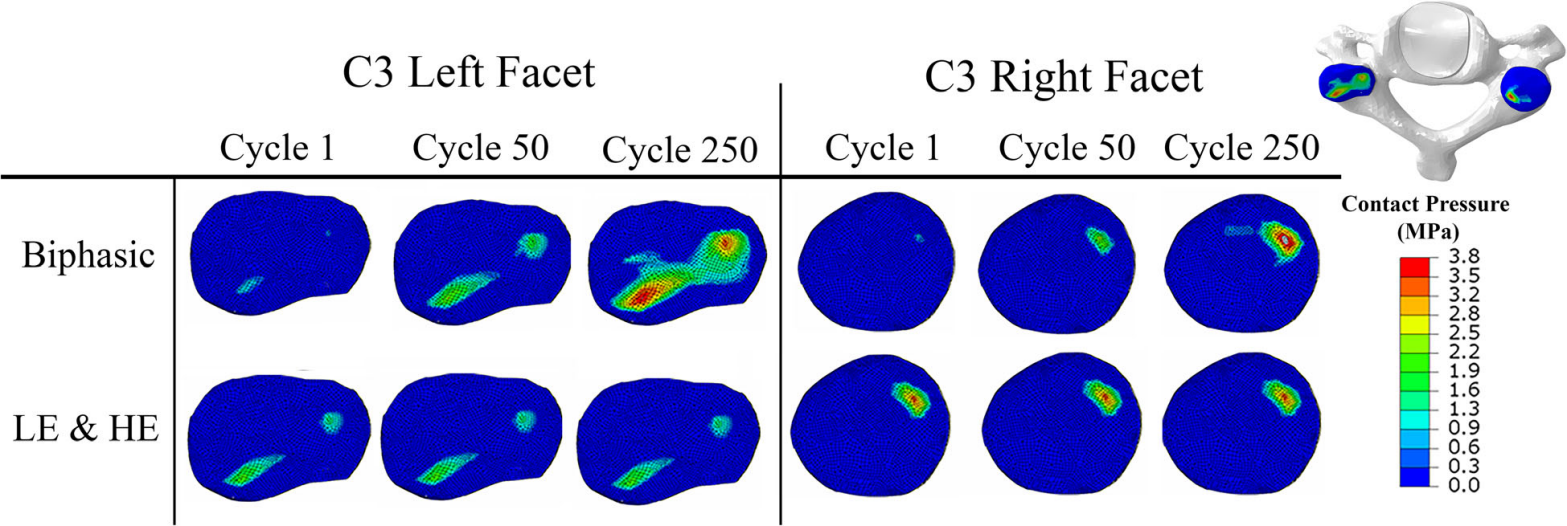
Facet joints’ contact pressure distributions predicted by the LE, HE, and Biphasic models at the peak of cycles 1, 50, and 250. The LE and HE models resulted in an approximately identical contact pressure distribution. The contact pressure of the Biphasic model increased with load repetition

**Fig. 4:**
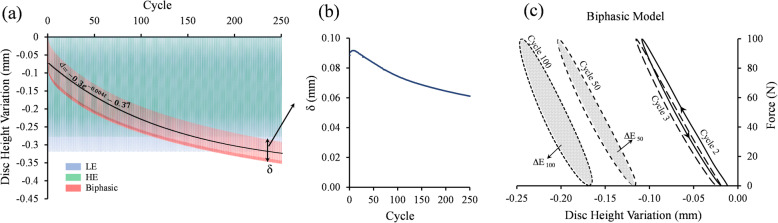
**a** The disc height variation over 600 s of cyclic compression obtained from the LE, HE, and Biphasic models. The disc had a purely sinusoidal height change in the LE and HE models. However, the disc height variation in the Biphasic model had a decaying sinusoidal oscillation with the amplitude of δ as shown in **b** about an exponential curve with the equation of ∆ =  − 0.3 *exp* (0.004*t*) − 0.37. **c** Representative force-disc height variation in cycles 2, 3, 50, 100. The dissipated energy (highlighted area) increased with the number of load repetitions. ∆E_*i*_ is the dissipated energy at cycle *i*

**Fig. 5:**
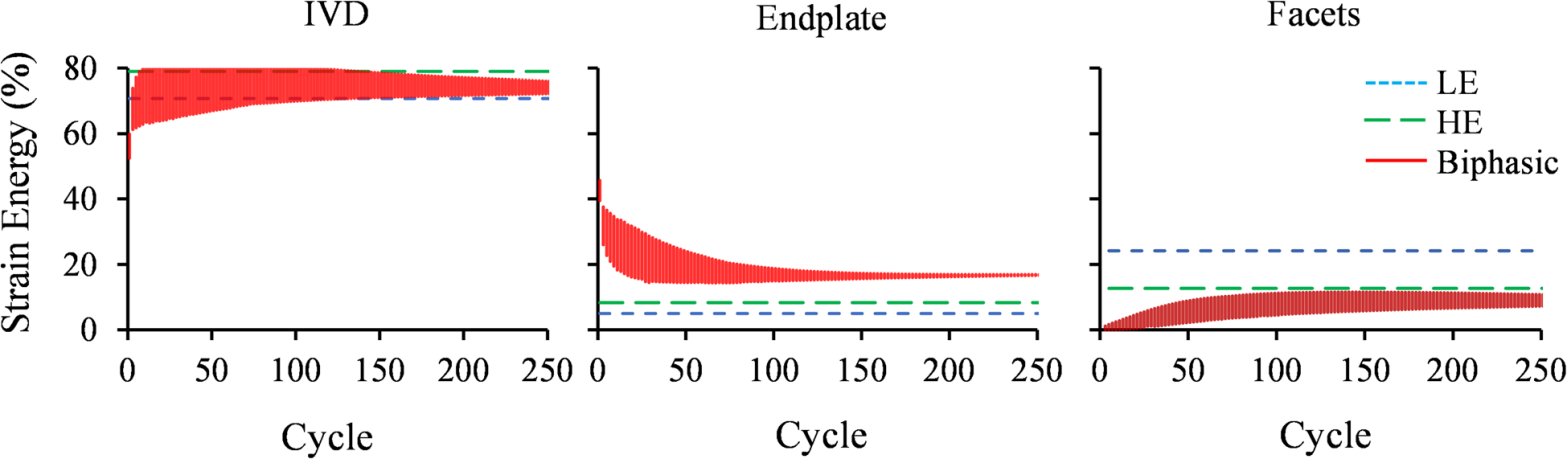
The predicted strain energy ratio stored in the IVD, endplates, and facets during the cyclic compression

The IVD stress profile obtained from the Biphasic model, including the IDP and compressive stress, showed a good agreement with experimental measurements [59] (Fig. 2). The linear region of the IVD stress profile in the Biphasic model results was longer compared to experimental results [59]. The hydrostatic stresses of the LE and HE models exhibited a similar stress profile but with magnitudes smaller than the experimental values. The performance of LE, HE, and Biphasic models under the cyclic compression was compared in terms of facet joints contact pressure (Fig. 3), and disc height variation (Fig. 4). The contact pressure area in the left facet joint was larger than the right one in all models (Fig. 3). The LE and HE models produced the same contact pressure profile at every cycle, while in the biphasic model, the final contact pressure profile was developed over the load repetitions (Fig. 3). Figure 4 illustrated the disc height variation during the cyclic loading. No permanent height decrease was observed in the results of the LE and HE models. However, the disc height decreased exponentially from one cycle to the next cycle in the Biphasic model. An exponential curve was fitted to the mean of disc height variations in Fig. 4a. The decaying oscillation of the disc height was illustrated in Fig. 4b. A schematic diagram of the hysteresis loop in the Biphasic model was illustrated in the force-displacement diagram of Fig. 4c, where the dissipated energy increased with repeated loads. The strain energy ratios of major load-bearing soft tissues, including the IVD, endplates, and facets ratios were illustrated in Fig. 5. The strain energy could be a representation of the load-sharing distribution. In the LE and HE models, {IVD, endplates, facets} contributions to load-sharing were {72, 5, 23%} and {79, 8, 13%}, respectively, which remained uniform from cycle 1 to the last cycle. However, in the Biphasic model, the load-sharing varied through cycles; facets’ contribution increased from 1% in cycle 1 to 12% in cycle 250. On the other hand, endplates’ contribution decreased from 40 to 17% from cycle 1 to 250, respectively. After cycle 250, 500 s of loading, the load-sharing distribution had less than 0.1% between two consecutive cycles in the Biphasic model (Fig. 5).

### Sagittal bending

**Fig. 6:**
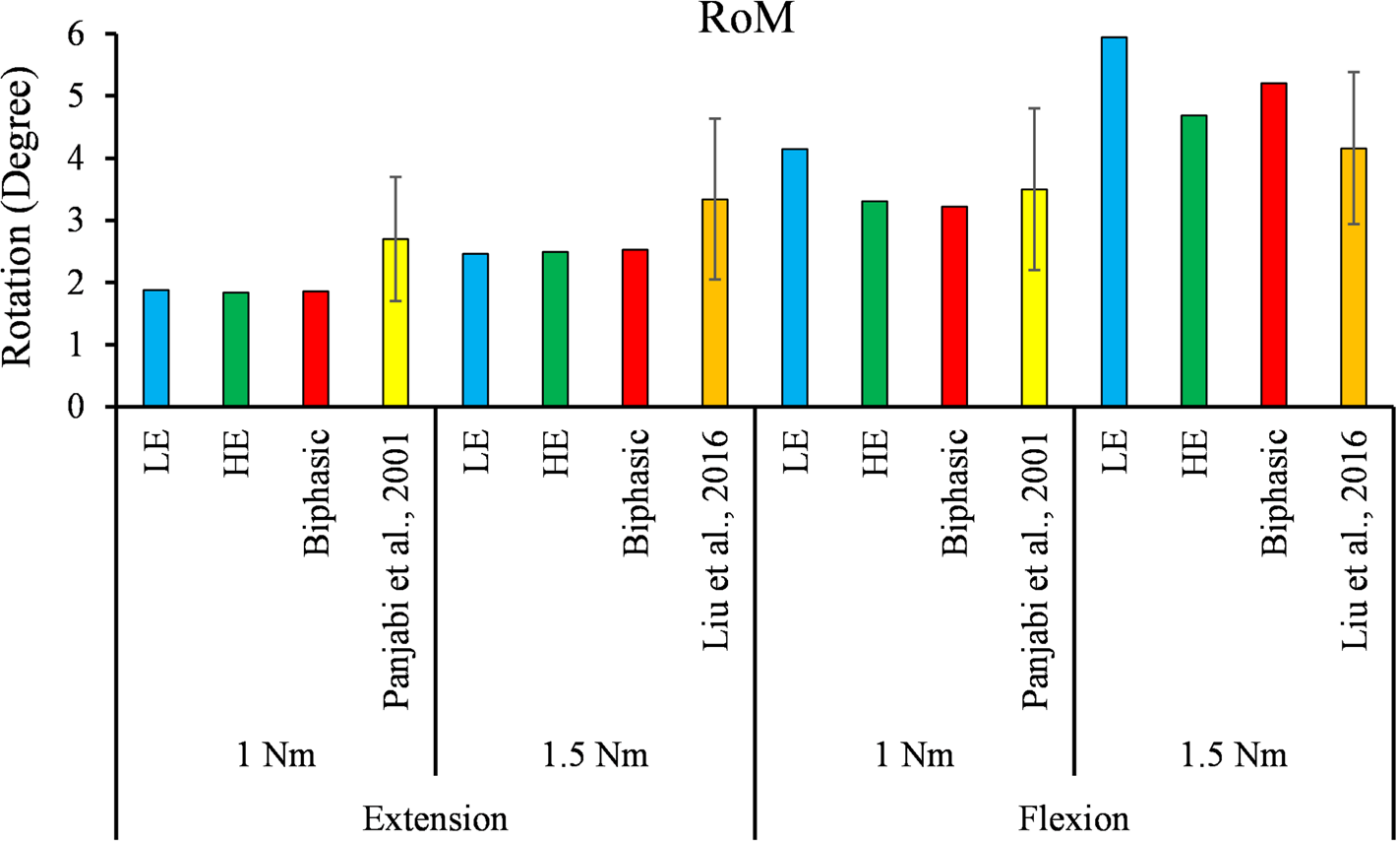
RoM (Degree) predicted by the Biphasic, LE and HE FEA models was compared with existing in vitro experimental data reported by Panjabi et al. [38], (1 Nm) and Liu et al. [63], (1.5 Nm) during flexion and extension

**Fig. 7:**
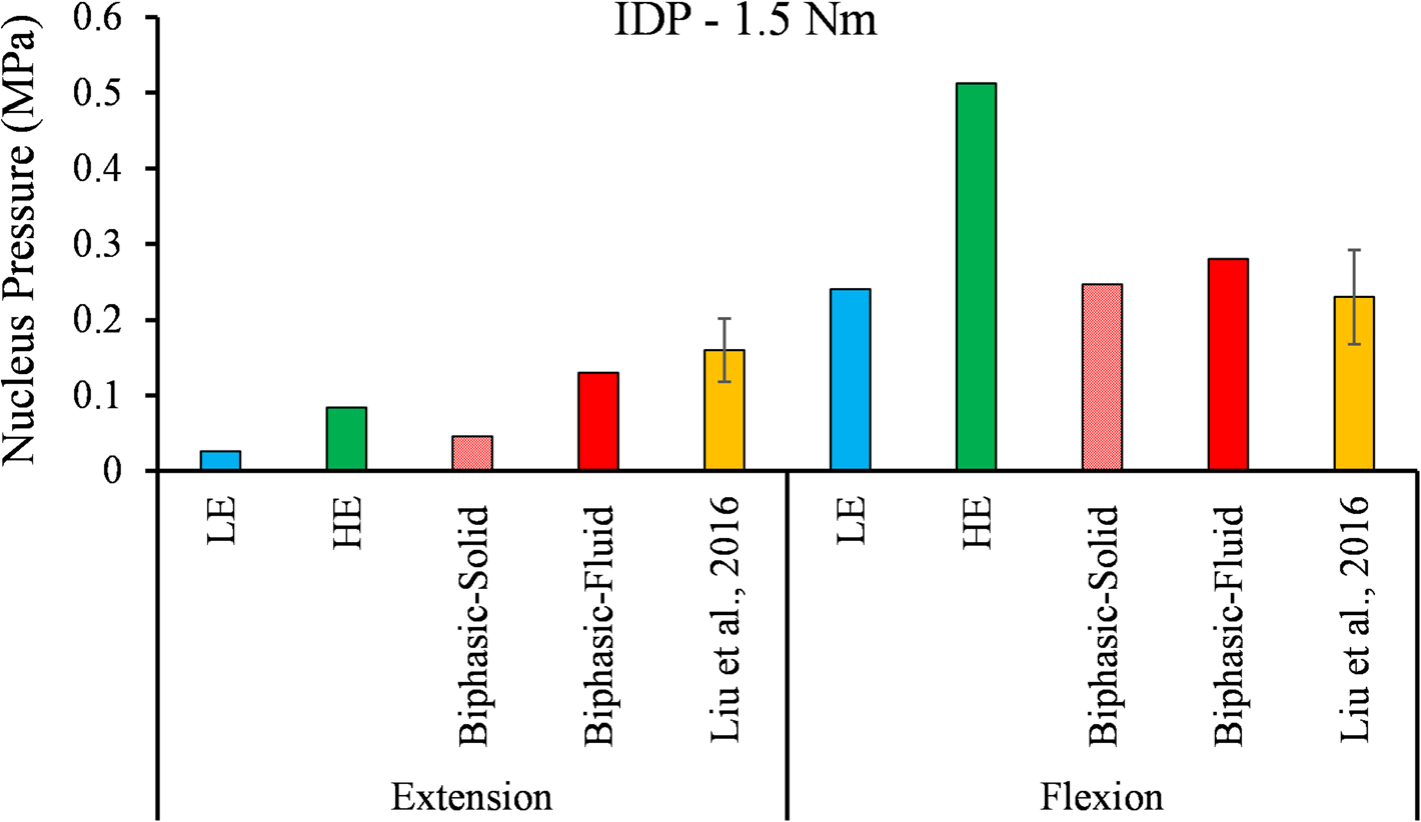
The predicted IDP was compared with the in vitro experimental data reported by Liu et al. [63], during 1.5 Nm flexion and extension

The FSU’s RoM predicted by all three models during 1 Nm and 1.5 Nm quasi-static flexion and extension were illustrated in Fig. 6. The predicted RoM in extension was almost identical in the three models. However, the RoM in flexion was higher in LE than the HE and Biphasic model. Fluid pressure in the NP predicted by the Biphasic model fell within 1 standard deviation (SD) of the IDP reported by Liu et al. [63] (Fig. 7). The LE and HE models did not consider the fluid phase, therefore the IDP was estimated by the hydrostatic pressure in these models and compared with the experimental IDP values (Fig. 7).

**Fig. 8:**
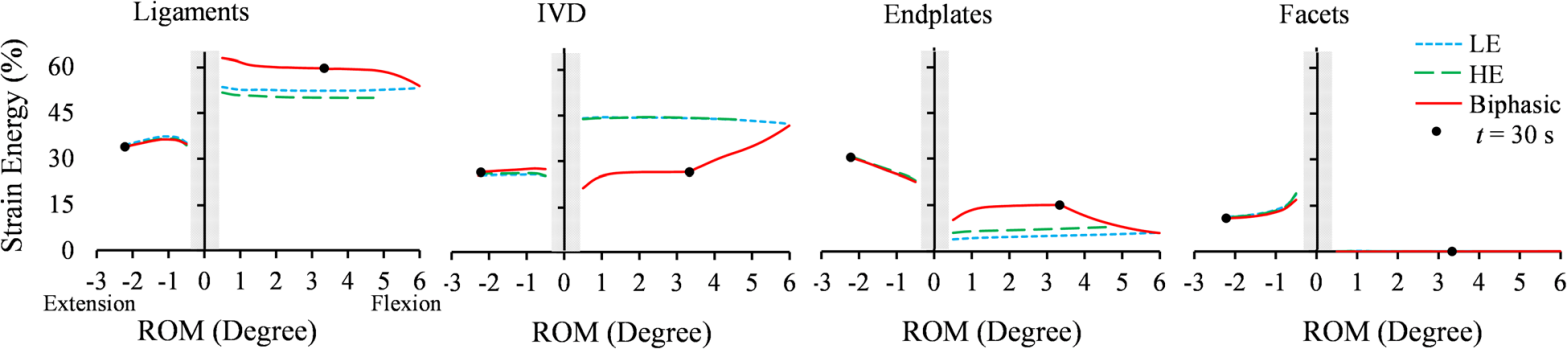
Strain energy distribution among FSU components predicted by the LE, HE, and Biphasic material models during 1.5 Nm flexion/extension that was applied in 30 s (loading phase), and kept for 10,000 s (creep phase). The solid dot (●) shows the start time point of the creep phase in the Biphasic model

**Fig. 9:**
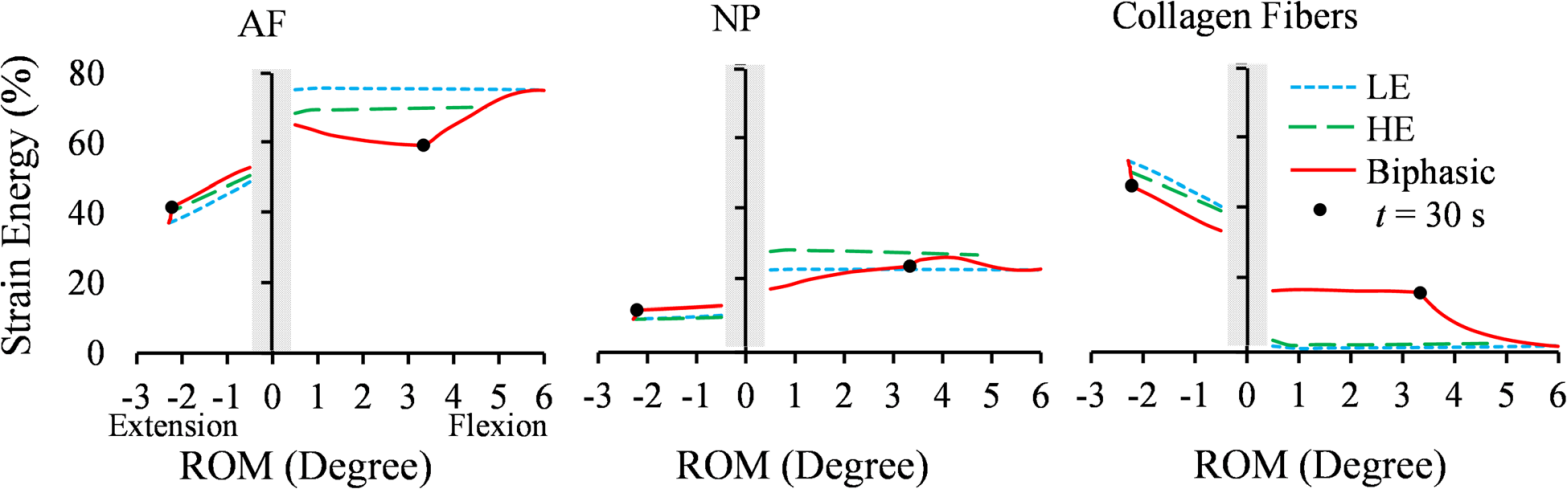
The strain energy contribution of AF, NP and collagen fibers during 1.5 Nm flexion and extension, applied in 30 s and kept for 10,000 s. The solid dot (●) represents the start time point of the creep phase at t = 30 s

The normalized strain energy ratio of each component represented its contribution to the FSU load-sharing. Major load-bearing components (ligaments, IVD, endplates and facets) were considered to study the effect of the IVD material model on the FSU load-sharing in Figs. 8 and 9. A 1.5 Nm flexion/extension was applied linearly in 30 s and kept for 10,000 s for creep (Fig. 8). The strain energy ratios within the first 0.5° of RoM were excluded because initial facet contact pressures introduced noises in results. During flexion, {ligaments, IVD, endplates, facets} carried {54, 40, 6, 0%}, {50, 42, 8, 0%}, and {60, 26, 14, 0%} of the FSU’s total strain energy in the LE, HE, and Biphasic models, respectively (Fig. 8). While the components’ contribution to load-sharing was uniform in the LE and HE model, they redistributed considerably in the Biphasic model during the creep phase. Facets did not show any contribution during the flexion (Fig. 8).

During extension, the strain energy ratio in the ligaments and IVD decreased with further rotations, while load-sharing contributions of the endplates and facets increased (Fig. 8). This load-sharing redistribution behaviour was observed in all models under extension with almost identical magnitudes of {34, 25, 30, 11%}.

Within the IVD, the AF had the largest contribution to the load-bearing during flexion (in the range of 60–80%), following by the NP and collagen fibers (Fig. 9). Under flexion, the IVD was compressed almost everywhere in the LE and HE models, resulting in a negligible collagen fibers contribution to the load-sharing (less than 1%). However, in the Biphasic model, a group of collagen fibers were extended due to the IDP and carried about 15% of the IVD strain energy (Fig. 9. right). During extension, collagen fibers’ contribution to IVD mechanical response reached up to 52% with rotation. Similar to Fig. 8., a redistribution of load-sharing occurred in the IVD components during the creep phase for the Biphasic model. In flexion, the load-sharing was transferred from collagen fibers to the AF and NP, increasing their contribution from 60 to 75% and 17 to 26%, respectively, at the creep phase. Whereas no change was observed in the LE and HE results during the loading and creep phases (Fig. 9). The AF and NP had more contribution to flexion than extension in all models.

## Discussion

The effect of LE, HE, and Biphasic IVD material models on the kinematics and kinetics responses of a C2-C3 model were investigated under flexion, extension, and cyclic compression. The solid phase in the LE and Biphasic models had identical material properties (Table 3), so any discrepancy between their mechanical responses would be caused by the solid-fluid interaction. The HE model was used to investigate whether the fluid-solid interaction effect in the FSU mechanical response could be described by material nonlinearity. The IVD material model affected the load-sharing, IDP and RoM under flexion and cyclic loadings more than the extension.

Before comparing the three LE, HE, and Biphasic models, their results were validated with experimental works for compression and flexion/extension loading conditions. The predicted IVD stresses under the 200 N ramp compression were compared with the experimental measurements in Fig. 2. The peak pressure at the NP and AF interface could be due to the change in permeability and elasticity modulus. From the linear part of the curve, the size of NP in [59] was smaller than the simulated NP in the present work. The disc pressure was approximately constant in the NP, and the mechanical response was governed mainly by the fluid pressure. This observation justified the use of cavity models in some studies to simulate the NP [27, 47, 64]. The predicted IDP in the NP under flexion and extension showed good agreement with the in vitro data measured by pressure transducers [63] (Fig. 7). The hydrostatic pressure of the NP in LE and HE models may not be a good representative of IDP in some loading conditions, such as extension (Fig. 7), however, it would be the most relevant parameter for IDP estimation in single-phase models. Peak IDP in extension was smaller than the flexion (Fig. 7). The FSU’ RoM predicted by all three models during 1 Nm and 1.5 Nm quasi-static flexion and extension were in good agreement with in vitro data [38, 63] except for the LE model during 1.5 Nm flexion, which was within two SD from the mean of experimental data (Fig. 6). The LE, HE, and Biphasic models predicted approximately identical RoMs (less than 2% difference) in extension (Fig. 6 - left), but their predictions diverged when the FSU was under flexion (Fig. 6 - right).

A cyclic compression was applied because it is a physiologically relevant loading condition. Moreover, under this dynamic loading condition, it was expected to obtain a clear discrepancy in the mechanical responses of the three IVD models. Overall, a repeated mechanical response was observed for the LE and HE models as these two models did not include any energy-dissipating element. Note that the viscoelastic ligaments were active only in extension. Contours of facets contact pressure (Fig. 3) during cyclic loads in the Biphasic model illustrated a growing load-bearing contribution from facet joints, which occurred mainly in the left facet. As a result, the contribution of facets in the strain energy increased from 1 to 12% in the Biphasic model after 250 cycles (Fig. 5). However, the LE and HE models resulted in an identical contact pressure contour at each cycle. The LE model was slightly softer than the HE model and resulted in about 12% more disc compression, as seen in Fig. 4a.

During a single compression cycle, the disc height variation was smaller in the Biphasic compared to the LE and HE models, indicating the importance of the IDP contribution to preventing excessive disc deformation under dynamic loading conditions. The LE/HE models predicted 0.32 and 0.27 mm disc heigh reduction at every cycle (Fig. 4a), respectively, while the disc heigh varied less than 0.1 mm at every cycle of compression (Fig. 4b). In the Biphasic model, the IVD did not have enough time during the unloading phase of cyclic compressions to recover its lost fluid, leading to an incremental disc height loss at every compression cycle that described a creep-like phenomenon. The exponential regression curve (*R*^2^ = 0.98) predicted a 0.37 mm permanent disc deformation after an infinite number of cyclic compressions. A corresponding value of 0.21 mm was obtained in an experimental study by Yoganandan et al. [65] (C4-C5 FSU, load amplitude = 150 N, *f* = 2 Hz). This permanent deformation in one spinal disc, when is cascaded to all vertebrae, may result in a 2–3 mm height loss [66]. The disc height variation had a decaying oscillation amplitude (*δ*) with time, started from *δ* = 0.09 mm in cycle 1 and decayed to a constant *δ* = 0.05 mm in cycle 250. The LE and HE models did not precisely predict the above discussed mechanical responses. Therefore, dynamic compression is an exemplary condition where the energy-conservative constitutive material models would fail to describe the IVD mechanical behaviour accurately.

Understanding spinal load-sharing is crucial in clinical studies [67]. In the present study, the strain energy of the FSU components was used to investigate the effect of IVD material models on the load-sharing response of FSU under flexion, extension and compression. In flexion, CL and PLL ligaments were the major load-bearing components in all three models, followed by IVD and endplates (Fig. 8). The ligaments and endplates strain energy ratios in the Biphasic model were about 10% greater than the corresponding values in LE and HE models. Reversely, the IVD strain energy ratio in the Biphasic model was 17% less than LE and HE models under flexion (Fig. 8). Facets contribution was negligible in flexion, yet reached 11% of total load-sharing in extension in all models (Fig. 8). The strain energy ratios were redistributed when creep started at t = 30 s in the Biphasic model (marked by a dot in Fig. 8); in extension, the role of facets in load-bearing increased, while the contribution of endplates and IVD decreased. This makes sense because when fluid exudes the IVD during the creep, it becomes deflated, and as a result, facets continue to carry more loads until their strain energy values reach the LE response at the end of the creep phase. The LE and HE models did not demonstrate this phenomenon.

The IVD components play distinct roles in various loading conditions. The AF was the major load-bearer in flexion, followed by NP and collagen fibers in all three models (Fig. 9). In extension, the AF was also the major load bearer at the beginning of loading, but with further extension, the collagen fibers showed more contribution than the AF. One major difference in the load-sharing response of the three models during flexion was the contribution of IVD collagen fibers; the LE and HE models predicted less than 2% strain energy ratio for collagen fibers, but this value was 20% in the Biphasic model. A higher contribution from collagen fibers under flexion reduced the contribution of AF from about 75% in the LE to 60% in the Biphasic model (Fig. 9. left). The IDP could cause the IVD volume increase in the Biphasic model and stretched collagen fibers during the loading phase. This could be considered an advantage of the Biphasic model, where an effective synergy among the IVD components was predicted which caused more uniform load-sharing distribution among components and reduced stresses. The discrepancy of load-sharing in the three models was less in extension than flexion. Apparently, in the creep phase, the IDP decreased gradually, and collagen fibers’ contribution reduced to about 2% as in the LE response. These results suggest that simulating a Biphasic IVD model is more important for flexion and compression than extension loading. A further investigation on considering the combined flexion/extension, lateral bending and axial compression may identify conditions that single-phase models would underperform. There are limitations to the presented FE simulation. Active musculature that exerts stabilizing forces on the spine was not simulated. The resultant force of spine muscles was summed by the applied loads in the present study. The series of multiple FSUs in the spine were simulated using an isolated C2-C3 FSU model, and the RoM was predicted within one SD from the mean of experimental measurements (Figs. 6 and 7). However, to simulate in vivo conditions more closely, a full spine model could be considered. Results of this study may be subject-specific; hence, sensitivity and parametric analyses could be conducted to investigate the reliability of the findings for different FSU sizes and material properties.

## Conclusion

The single-phase IVD material models failed to describe the long-term IVD height decrease under cyclic loading. The contact area and contact pressure of facet joints were different in the Biphasic and LE/HE models. While a small difference was observed in the mechanical response of C2-C3 FSU under extension, the Biphasic and LE/HE models predicted distinct load-sharing during the loading phase of flexion. However, when the static condition is reached, Biphasic and LE models predicated the same mechanical response. These findings include that applying simpler material models, such as linear elastic, is justifiable when the spine is under quasi-static extension. However, more accurate material models, such as biphasic, are required for modeling intervertebral disc when the spine is under dynamic compression and flexion.

## Data Availability

Data sharing is not applicable to this article as no datasets were generated or analysed during the current study. The spine model used and analyzed in the current study is available upon reasonable request to Dr. Amin Komeili (AK; akomeili@uoguelph.ca).

## Abbreviations

LE: Linear Elastic
HE: Hyperelastic
NP: Nucleus Pulposus
AF: Annulus Fibrosus
IVD: Intervertebral Disc
IDP: Intradiscal Pressure
FSU: Functional Spinal Unit
RoM: Range of Motion
FE: Finite Element
CL: Capsular Ligament
ISL: Interspinous Ligament
ITL: Intertransverse Ligament
LF: Ligamentum Flavum Ligament
PLL: Posterior Longitudinal Ligament
SSL: Supraspinous Ligament
